# 

**Published:** 1893-10

**Authors:** 


### CONTENTS:

ORIGINAL ARTICLES: PAGE

Horse Show—Columbian Exhibition. By A. H. Godfrey .......... 193
Extirpation of a Tubo-Ovarian Cyst. By Dr. R. A. Archibald .......... 224
Scouring in Calves. By John M. Parker .......... 227
Bovine Tuberculosis. By John M. Parker .......... 235

EDITORIAL:

Apology .......... 246
United States Veterinary Medical Association .......... 247

COMMUNICATION:

Iatrol .......... 260

SOCIETY PROCEEDINGS:

Pennsylvania State Veterinary Medical Association. Annual Meeting .......... 261
Pennsylvania State Veterinary Medical Association " " .......... 263
Pennsylvania State Veterinary Medical Association. Semi-Annual Meeting .......... 274
California State Veterinary Medical Association .......... 276
Illinois State Veterinary Medical Association .......... 278
Kansas State Veterinary Medical Association .......... 279

Obituary: Robert Jennings .......... 280

